# Understanding College Students’ Healthcare Avoidance: From Early Maladaptive Schemas, through Healthcare Institutional Betrayal and Betrayal Trauma Appraisal of Worst Healthcare Experiences

**DOI:** 10.3390/healthcare12111126

**Published:** 2024-05-31

**Authors:** Pedram J. Rastegar, Jennifer Langhinrichsen-Rohling

**Affiliations:** Health Psychology PhD Program, University of North Carolina at Charlotte, 9201 University City Boulevard Colvard, Charlotte, NC 28223, USA; jlanghin@charlotte.edu

**Keywords:** healthcare avoidance, healthcare institutional betrayal, early maladaptive schemas, trauma appraisal

## Abstract

Understanding healthcare avoidance among college students is critical. In this study, we consider two broad cognitive contributors to greater healthcare avoidance: specific early maladaptive schema and negative appraisals of students’ prior worst healthcare experiences. From schema theory, we proposed college students holding greater levels of two early maladaptive schema (disconnection/rejection and impaired autonomy/performance EMS) would be more likely to appraise their problematic healthcare experience as both containing healthcare institutional betrayal (HIB) behaviors and as traumatic and betrayal-inducing; both EMS and these appraisals would predict healthcare avoidance. Using a cross-sectional survey in a large, diverse college student sample (*n* = 1383, 61.1% female, 18.9% African American, 7.2% Asian, 6.4% Hispanic/Latino), as predicted, both EMS were significantly related to healthcare avoidance. Furthermore, a sequential mediation model was supported, indicating students holding greater EMS of disconnection/rejection or impaired autonomy/rejection reported more HIB in their worst healthcare experience, and appraised that experience as more betraying. Taken altogether, this model accounted for 23% of the variance in students’ reports of healthcare avoidance. Core beliefs formed early in life may be a foundational lens through which potentially traumatic healthcare experiences are processed in ways that can impact emerging adults’ future healthcare engagement. Findings also support the importance of addressing HIB actions and repairing trauma appraisals accrued during problematic healthcare experiences to prevent healthcare avoidance by emerging adults.

## 1. Introduction

Healthcare avoidance, which in this study is broadly defined as avoiding or delaying seeking needed medical treatment, is a prevalent and serious problem. Studies suggest at least one-third of various groups of respondents report avoiding medical care [1,2]. Delay or avoidance in seeking healthcare can have deleterious consequences including worse prognosis as well as poorer response to treatment [2,3]. For example, delay in seeking treatment for stroke contributes to cardiovascular morbidity burden [4]. Conversely, diagnosis and treatment at presymptomatic phases of cancer diagnosis are related to a higher survival rate [5].

College students’ healthcare avoidance may constitute preventable behavior. College students comprise an important patient population as many are at a critical developmental juncture with regards to their health, safety, injury, and risk-taking behaviors [6]. Developmentally, these emerging adults are generally experiencing increased autonomy and individuation from their family of origin, while taking on greater management of their own healthcare, including, in some cases, seeking or managing care for long-standing or chronic mental and physical health concerns [7]. Some are making the transition from pediatric or family-managed healthcare to adult or autonomous healthcare in ways that will establish their adult healthcare routines and their life-long health-related decision-making processes and engagement strategies.

College students may also be at particularly high risk for healthcare avoidance. Recently, it was estimated that about 62 percent of college students endorsed engaging in healthcare avoidance [8]. Therefore, understanding predictors of healthcare avoidance in this population is critical. This study addresses that gap.

### 1.1. BITTEN Model

Predictors of healthcare avoidance have been described within the BITTEN framework, which centers around the patient experience [9]. According to BITTEN, a patient’s future healthcare engagement is predicated on several historical factors and experiences. Two are the focus of the current study. First, some patients have a family of origin history that can include a variety of adverse childhood experiences. According to schema theory, these historical experiences can generate a variety of maladaptive beliefs or schema that persist into adulthood. These schemas can, in turn, influence how healthcare experiences are appraised and processed. Second, the BITTEN framework postulates that patients’ past problematic healthcare experiences also influence their likelihood of current and future engagement with the healthcare system [9]. Specifically, BITTEN suggests that if a patient has experienced healthcare institutional betrayal (HIB) during a problematic healthcare encounter and/or appraised the healthcare system as unworthy of trust (betrayal trauma appraisal), they will be less likely to return to that system for care and more likely to avoid healthcare engagement in general. In this model, healthcare institutional betrayal or HIB is defined as a system-level transgression committed against an individual who is reliant on that institution for support or care [10].

Multiple recent studies in college student populations provide support for using the BITTEN framework to understand healthcare avoidance. For example, the link between past experiences of HIB and expected future healthcare avoidance has been demonstrated in this population [11]. Additionally, other forms of medical trauma, such as receiving nonconsensual surgery, have been associated with healthcare avoidance [12] as would be expected per BITTEN. Conversely, as also predicted by BITTEN, prior positive healthcare experiences, occurring during childhood, have been shown to predict better healthcare behaviors and greater healthcare engagement in adulthood, as measured by items such as attending doctor visits regularly [13].

However, few existing studies have explored the various aspects of the BITTEN framework working conjointly. As an exception, one previous study relied on the BITTEN framework to test if childhood trauma would predict healthcare avoidance and if, as expected, this relationship would be mediated by trust in healthcare providers [11]. In this previous publication, HIB was considered as a moderator. Supporting the BITTEN model, the authors found that childhood trauma significantly predicted healthcare avoidance and that, as predicted theoretically, trust in healthcare providers partially mediated this relationship. Lastly, childhood trauma had stronger associations with trust in healthcare providers when participants reported higher levels of HIB [11].

While this prior research provides broad support for the BITTEN model as theorized, an understudied feature of the framework is whether there are key cognitive processes underlying the proposed BITTEN relationships. For instance, childhood trauma is known to influence healthcare avoidance, but the cognitive structures underlying this relationship are unknown. Theoretically, identifying specific cognitive processes could facilitate the generation of potentially modifiable intervention targets. For example, a set of beliefs that are a persisting marker of negative or adverse family origin experiences are the endorsement of early maladaptive schemas (EMS). To date, however, little research has explored how these particular cognitive processes could further explicate the BITTEN model in ways that might enhance our understanding of, and ability to prevent, patients’ healthcare avoidance. This study is designed to fill that gap.

Specifically, early maladaptive schema, or EMS, have been defined as dysfunctional beliefs about the self and others that are formed in childhood in contexts that include trauma or adverse childhood experiences [14]. Schema theory posits that certain damaging early childhood experiences with trusted caregivers can generate maladaptive core themes about ourselves, others, and the world [14]. These maladaptive core beliefs can then keep re-emerging throughout life, resulting in chronic, moment-to-moment engagement in characteristic and often unproductive coping strategies and emotions in response to challenging events, known as schema modes. These schema modes, because they develop early in life, become familiar, and are often experienced as valid interpretations of events. They can also lie dormant until they are activated to provide an organizational frame for coping with particularly challenging situations [15].

The challenging situation of focus in the current study is college students’ worst previous healthcare experience. Two schemas are thought to be particularly relevant to experiencing HIB during one’s worst healthcare experience: those from the impaired autonomy and performance domain and those from the disconnection and rejection domain. The impaired autonomy and performance EMS domain includes beliefs about self-vulnerability as well as concerns about one’s ability to be independent or successful [14]. Examples of impaired autonomy and performance schema include pervasive thoughts of being a failure, and excessive worry about vulnerability to harm and illness [14]. Given that patients need to rely on the healthcare system for care as well as to obtain critical information about their body, greater thoughts of self-vulnerability, increased worry about harm, and lack of confidence in successful decision-making should predict greater susceptibility to perceiving HIB during a challenging healthcare experience.

The EMS disconnection and rejection domain has been broadly defined to include thoughts and beliefs that one will not be accepted or respected by others [14]. Disconnection and rejection schemas include the belief that one will be abandoned, concerns that one will be socially isolated or excluded, and beliefs that one should be mistrustful of others [14]. Theoretically, holding beliefs that one will not be well cared for by others, via rejection or abandonment, should also predict greater likelihood of perceiving the healthcare system as non-supportive and rejecting, which should be associated with greater HIB as well as with healthcare avoidance directly. In broad support of this supposition, a recent meta-analysis of 49 studies indicated that both the impaired autonomy and performance and disconnection and rejection EMS domains had stronger associations with interpersonal problems than EMS from other domains [16].

Given this, we predict that beliefs of failure, vulnerability to harm and illness, and mistrust/abuse associated with the impaired autonomy and performance and disconnection and rejection EMS domains, if held by a patient, would activate during a patient’s worst health care experience. Once activated, these beliefs could both promote an attentional bias toward noticing HIB and could also result in receiving lower-quality healthcare from providers (e.g., if the patient has a trauma response that is not anticipated nor reacted to well from their providers). Consistent with this reasoning, we predict that college students holding greater levels of either of these two schemas will report more HIB occurring during their worst healthcare experience. They will also appraise their worst previous healthcare experience as more traumatic.

We further assert that reporting acts of HIB in one’s worst healthcare experience is a critical part of the process that leads to healthcare avoidance. Greater HIB is associated with a host of negative outcomes including greater psychological harm to the individual, increased likelihood of traumatic reactions and/or stress related diagnoses, and on-going distrust and potential avoidance of that healthcare institution and those who represent it, post trauma [10]. HIB includes acts of commission and omission such as the following: the mismanagement of sensitive patient health information or the failure to follow routine patient safety or privacy procedures [17]. In and of itself, HIB is expected to result in greater healthcare avoidance.

While HIB is a marker of problematic system-level acts that occurred during a healthcare experience, currently, the most widely used measure of HIB (IBQ-H [17]) does not assess for betrayal appraisals of the experience. Yet, those appraisals are central to the BITTEN framework as a mechanism for understanding patients’ future health-related needs, expectations, and behaviors, including healthcare avoidance. In the current study, we propose that both greater EMS and HIB will increase the likelihood of healthcare avoidance through trauma appraisal. Trauma appraisal has been defined as the degree to which one negatively evaluates their thoughts, feelings, and behaviors after a potential traumatic event [18].

In terms of healthcare avoidance, betrayal trauma theory suggests that traumatic events perpetrated by an individual or institution who was trusted or depended upon will result in a more negative impact for the survivor [19,20]. Although the theory does not require the individual to appraise the acts as betrayal, this type of judgment has been linked to the development of other problems including post-traumatic stress disorder (PTSD) [20] and has also been shown to be a mechanism between EMS and PTSD outcomes. For example, negative traumatic appraisals of shame mediated the relationship between the impaired autonomy and performance EMS domain and PTSD symptom severity, as well as between the disconnection and rejection schema EMS domain and PTSD symptom severity [21]. Based on this previous work, we expected that betrayal trauma appraisals (BTA) would mediate the pathway between EMS and healthcare avoidance. Specifically, we predict that individuals who have long-standing and pre-existing beliefs of failure, mistrust of others, and fears about being abandoned (i.e., impaired autonomy and performance and disconnection and rejection domains of EMS) will be more likely to report HIB during their worst healthcare experience, which would then increase appraisals of betrayal during this experience. These factors (EMS, HIB, and betrayal trauma appraisal) will sequentially predict healthcare avoidance in keeping with predictions generated from the BITTEN model.

### 1.2. Current Study

**Figure 1:**
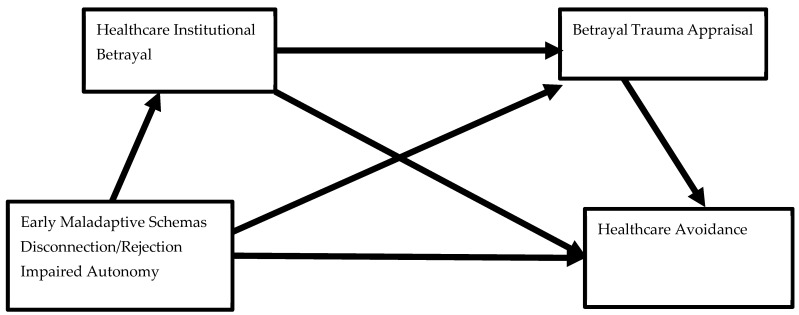
Conceptual model of early maladaptive schema predicting healthcare disengagement/avoidance sequentially through the experience of healthcare institutional betrayal and betrayal trauma appraisal during one’s worst previous healthcare experience.

In summary, the present study utilizes the BITTEN model as a framework to understand how enduring EMS (impaired autonomy and performance and disconnection/rejection), HIB (system-level behaviors during one’s worst healthcare experience), and betrayal trauma appraisal (betrayal appraisals during one’s worst healthcare experience) will conjointly predict healthcare avoidance in college students. Theoretically, we expect that a young adult’s worst healthcare experience would be experienced as a challenging situation or a trigger in those holding certain maladaptive schemas, which would then activate a cascade of appraisals including greater endorsement of HIB acts which would, in turn, generate a greater likelihood of appraising one’s worst healthcare experience as a betrayal. Self-reported gender and race were retained as covariates in these analyses given previous empirical research demonstrating greater self-reported HIB [22] and healthcare avoidance [23,24] in People of Color as well as gender differences on these measures (See Figure 1).

## 2. Methods

### 2.1. Procedures

The study sample consisted of 1383 undergraduate students who were recruited through online research subject pools serving psychology departments located in two large diverse public universities in the southeast United States. These universities were utilized because they were associated with the authors of this study, and they are known to have a diverse student body. After providing informed consent, participants completed online questionnaires assessing their early maladaptive schemas. They then were asked to focus on their self-selected worst health care experience. They then reported HIB acts experienced during this event, and their appraisal of betrayal about this experience. Participants were also assessed for their likelihood of future healthcare avoidance. Participants earned research credit for their introductory psychology courses by completing this study; alternate activities and studies to obtain credit were available. All study procedures were approved by the relevant institutional review boards at the two universities and ethical practices were followed throughout. Data collection occurred from 2019 to 2020 and the median time to complete the instrument was approximately 20 min.

### 2.2. Measures

#### 2.2.1. Demographics

Participants self-reported their gender by choosing one of the following: female, male, prefer not to say, prefer to self-describe, or prefer not to answer. Participants also self-reported their racial identity by choosing White, Black/African American, Hispanic/Latino, Asian, American Indian or Alaskan Native, Native Hawaiian, Other, or prefer not to answer. Lastly, participants reported their age and current year in school.

Full details on sample characteristics and descriptives of study variables are provided in Table 1 and Table 2. Overall, the sample was diverse and representative of the universities from which they were drawn. However, participants were majority White (60%, *n* = 823) and female (61%, *n* = 845). Many in the sample were in their first year at college (48%, *n* = 661) and the mean age was 19.86 years old (*SD* = 3.64).

#### 2.2.2. Early Maladaptive Schemas

Impaired Autonomy and Performance. To create this domain, participants completed a truncated version of the Young Schema Questionnaire 3 Short Version [25]. Items were averaged to create the subscales that comprise the impaired autonomy and performance domain. These subscales include the following: dependence/incompetence, vulnerability to harm and illness, and failure. However, enmeshment/underdeveloped self, which is a facet of impaired autonomy and performance, was not assessed in the current study. The subscales of dependence/incompetence, vulnerability to harm and illness, and failure were then summed to create an overall impaired autonomy performance domain score. Higher scores indicate greater endorsement of impaired autonomy and performance beliefs. This domain had strong internal consistency (α = 0.89).

Disconnection and Rejection. Participants completed an abridged version of the Young Schema Questionnaire 3 Short Version [25]. In order to create a disconnection and rejection domain total score, responses to individual items of the Early Maladaptive Schema Long Form were averaged to create the following five subscales: abandonment, mistrust/abuse, emotional deprivation, defectiveness/shame, and social isolation. These five subscales make up the EMS disconnection and rejection domain. These subscales were then summed to create an overall disconnection and rejection domain score; higher scores indicate greater endorsement of disconnection and rejection Beliefs. This domain showed excellent reliability (α = 0.95).

#### 2.2.3. Worst Healthcare Experience

Participants were asked the following “Now, think about your WORST/MOST FRIGHTENING healthcare experience when answering the following questions. Describe why you sought healthcare in the first place when you had your WORST healthcare experience”. Participants were provided with an open-ended text box to provide answers to why they sought healthcare in the first place. They were then administered the following measures to determine if their worst healthcare experience included acts of healthcare institutional betrayal, the extent that experience was appraised as traumatic, and the degree to which they had engaged in healthcare avoidance since that experience.

#### 2.2.4. Institutional Betrayal

Participants completed the Institutional Betrayal Questionnaire-Health (IBQ-H) [17], which is a 12-item measure that asked about behaviors experienced during the participant’s worst health care experience. Participants responded “yes” or “no” to questions about whether the healthcare organization performed an act that contributed to their negative healthcare experience (e.g., did they mishandle your protected personal information or create an environment in which unpleasant health experiences were normalized). Items were then summed to create a composite HIB score with higher scores indicating more acts of HIB endorsed during one’s worst healthcare experience. The internal consistency of this measure in our study was acceptable (α = 0.67) [26].

#### 2.2.5. Trauma Appraisal Questionnaire

Participants completed the betrayal subscale of the larger 54-item Trauma Appraisal Questionnaire measure (TAQ) [18]. The TAQ-Betrayal subscale is a 7-item measure assessing participants’ agreement with thoughts and feelings in response to a traumatic incident from the past. For this study, we adapted the questionnaire to ask how much participants agreed with the thoughts and feelings depicted in the item in response to their worst health care experience. Items were worded such as, “I felt betrayed” or “The person who was supposed to be closest to me hurt me the most”. Participants rated how much they agreed with each statement on a 5-point scale from strongly disagree (1) to strongly agree (5). Items were summed to create an overall betrayal trauma appraisal (BTA) score with higher scores indicating greater appraisal of traumatic betrayal during their worst healthcare experience. This measure had excellent internal consistency in our study sample (α = 0.94).

#### 2.2.6. Healthcare Avoidance

To measure healthcare avoidance, the authors generated three items that assessed avoidance from healthcare. Participants were asked to what extent they have avoided seeking medical care, been fearful of seeking medical care, and/or changed their healthcare approach (e.g., only seeking medical care if symptoms are severe) since their worst healthcare experience. Participants rated their level of avoidance on a 4-point scale ranging from not at all (1) to very much (4). Items were summed and higher scores indicate higher levels of healthcare avoidance. This measure had good internal consistency in our study sample (α = 0.84).

### 2.3. Planned Analyses

#### 2.3.1. Preliminary Analysis

All data were analyzed with SPSS Version 28. Descriptive analyses were conducted to ensure data were normally distributed and had acceptable variance. Pearson’s correlations were conducted to examine relationships among study variables. Gender and race were used as covariates to control for group differences in self-reported HIB [22] and healthcare avoidance [23,24]. However, since some groups of race and gender were very small, these variables were dichotomized (e.g., 0 = female and 1 = male; 0 = White and 1 = People of Color). Data from all participants who completed at least 80% of the items for that subscale were retained. To account for missing data for the EMS subscales, participants’ completed responses were used to generate interpolated total scores.

#### 2.3.2. Primary Analyses

To test for serial mediation, Process Macro Model 6 was used in SPSS Version 28. Bootstrapping was conducted to compute confidence intervals. To reduce model complexity and multicollinearity, the two early maladaptive schema domains were considered in separate models. Race and gender, coded dichotomously, were included as covariates. Females and Whites were used as the reference groups. To detect mediation, we examined the direct relationship of EMS with HIB, the direct relationship of EMS and HIB with betrayal trauma, and the indirect relationship of EMS and both mediating variables with healthcare avoidance. Mediation was detected if the confidence intervals of the indirect effect did not include zero [27].

## 3. Results

Correlations among study variables are provided in Table 3. As predicted, healthcare avoidance was significantly correlated with all study variables including both EMS subscales (*r*’s = 0.25 to 0.28, *p*’s *<* 0.001), HIB (*r* = 0.37, *p* < 0.001) and betrayal trauma appraisal during one’s worst healthcare experience (*r* = 0.37, *p* < 0.001).

Furthermore, both the impaired autonomy and performance and disconnection and rejection EMS domains were significantly associated with greater endorsement of HIB; however, the obtained effect sizes were small (*r* = 0.14 and *r* = 0.19, *p*’s *<* 0.01). Moreover, the impaired autonomy and performance (*r* = 0.22), and disconnection and rejection schema (*r* = 0.21) were also associated with betrayal trauma appraisal. Finally, the largest correlation obtained was between reports of HIB acts and betrayal trauma appraisal during the worst healthcare experience (*r* = 0.43). Identifying as a Person of Color was associated with lower levels of reported HIB but greater endorsement of disconnection and rejection schemas, both with small effect sizes. Gender was significantly correlated with all study variables except for disconnection and rejection EMS. Males reported lower levels on these measures, with all effects being small in magnitude.

### 3.1. Primary Results

Assumption tests for multiple regression were conducted. Histograms of the standardized residual followed a normal distribution. Further P-P plots of the residual of the regression show a linear relationship between our independent variables and our dependent variable. The Durbin–Watson test was under 2.5 which indicates independence of residuals. There was no multicollinearity as all correlations were under 0.80. Lastly, the assumption of homoscedasticity was violated. However, evidence suggests that regression analyses are robust to violations of homoscedasticity [28].

### 3.2. Impaired Autonomy Performance

**Figure 2:**
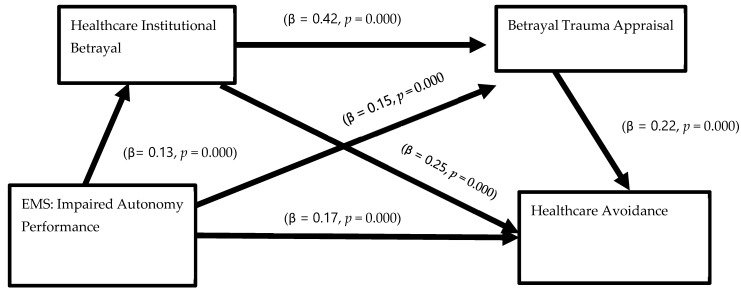
Serial mediation pathway: impaired autonomy performance predicts healthcare avoidance through HIB and trauma appraisal.

Table 4 reports the results generated from the mediation analyses of impaired autonomy and performance EMS on healthcare avoidance through healthcare institutional betrayal and betrayal trauma appraisal. Our initial model predicting HIB was significant (*R*^2^ = 0.05, *p* = 0.000) and explained 5% of the HIB variance. Higher levels of impaired autonomy and performance EMS predicted higher levels of HIB (β = 0.13, *p* = 0.000). Males, (β = −0.10, *p* = 0.000), and People of Color, (β = −0.17, *p* = 0.001) self-reported lower levels of HIB compared to females and Whites during their worst healthcare experience. Next, our second model predicting betrayal trauma appraisal was also significant and explained 22% of the variance. Both impaired autonomy and performance EMS (β = 0.15, *p* = 0.000) and HIB (β = 0.42, *p* = 0.000) predicted higher levels of betrayal trauma appraisal. Neither race nor gender significantly predicted betrayal trauma appraisal in this model. Finally, the full model predicting healthcare avoidance was significant and explained 23% of the variance seen in the model. Higher levels of impaired autonomy and performance EMS (β = 0.17, *p* = 0.000), HIB (β = 0.25, *p* = 0.000), and betrayal trauma appraisal (β = 0.22, *p* = 0.000), all predicted higher levels of healthcare avoidance. Participants who identified as a Person of Color (β = 0.06, *p* = 0.039) reported higher levels of healthcare avoidance. Lastly, as predicted via BITTEN, there was a significant indirect relationship of EMS and both mediating variables (HIB and betrayal trauma appraisal) on healthcare avoidance based on their non-zero confidence intervals. In order words, impaired autonomy and performance EMS predicted healthcare avoidance sequentially through reports of HIB and betrayal trauma appraisal in one’s worst healthcare experience. See Figure 2 illustrating the significant pathways.

### 3.3. Disconnection and Rejection

**Figure 3:**
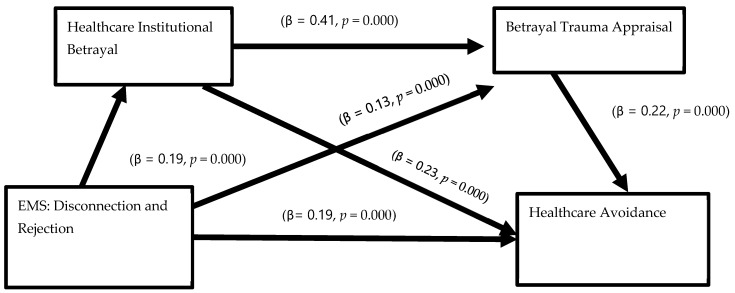
Serial mediation model: disconnection and rejection predicts healthcare avoidance through HIB and trauma appraisal.

Table 4 depicts the results of the mediation analyses of disconnection and rejection performance EMS on healthcare avoidance through HIB and betrayal trauma appraisal. Our initial model predicting healthcare institutional betrayal was significant (*R*^2^ = 0.07, *p* = 0.000). Higher levels of disconnection and rejection EMS predicted higher levels of HIB (β = 0.19, *p* = 0.000). As before, males, (β = −0.17, *p* = 0.000), and People of Color, (β = −0.12, *p* = 0.000) self-reported lower levels of HIB compared to females and Whites during their worst healthcare experience. In the second step, our model predicting betrayal trauma appraisal was also significant (*R*^2^ = 0.21, *p* = 0.000) and explained 21% of the variance. Both disconnection and rejection EMS (β = 0.13, *p* = 0.000) and HIB (β = 0.41, *p* = 0.000) predicted higher levels of betrayal trauma appraisal. Neither race nor gender significantly predicted betrayal trauma appraisal. Next, the full model predicting healthcare avoidance was significant (*R*^2^ = 0.23, *p* = 0.000) and explained 23% of the variance seen in the model. Disconnection and rejection EMS (β = 0.19, *p* = 0.000), HIB (β = 0.23, *p* = 0.000), and betrayal trauma appraisal (β = 0.22, *p* = 0.000) were all retained in the model to predict higher levels of healthcare avoidance. Again, males (β = −0.06, *p* = 0.032) reported lower levels of healthcare avoidance compared to females. Lastly, there was a significant indirect relationship of EMS and both mediating variables on healthcare avoidance based on their non-zero confidence intervals. In order words, disconnection and rejection EMS predicted healthcare avoidance sequentially through HIB and betrayal trauma appraisal. See Figure 3 illustrating the significant pathways.

## 4. Discussion

The BITTEN model centers the patient within the healthcare experience and identifies past experiences that, if left unaddressed, are likely to influence current and future healthcare related behavior. More specifically, the BITTEN model suggests that a patient’s future healthcare engagement can be predicted by prior trauma history such as adverse childhood experiences as well as by previous experiences of HIB. Theoretically, adverse childhood experiences and HIB may interact to trigger trauma symptoms which can then influence how the patient experiences the healthcare system and how the healthcare system treats the patient. Conjointly, these experiences can then impact a patient’s future healthcare engagement.

In the current study, we focused on specific cognitions that may be driving college students’ healthcare avoidance. The focus on healthcare avoidance among college students is essential because college students are at high risk for this behavior [8]. Additionally, it is imperative to understand risk factors for healthcare avoidance as delay or avoidance in healthcare may lead to worse prognosis as well as poorer response to treatment [2]. Furthermore, it is important to understand cognitive processes that underlie healthcare avoidance as research suggests that cognitive processes can influence current health behaviors [2] as well as engagement in health prevention activities. They are also potentially modifiable.

In keeping with the BITTEN framework, our proposed models accounted for 23% of the variance in healthcare avoidance and all elements of the model were retained as predictors. Namely, early maladaptive schemas directly predicted health care avoidance. Additionally, we found that the pathway to avoidance was sequentially explained through HIB and betrayal trauma. In other words, early maladaptive schemas are increasing healthcare avoidance through increasing endorsement of HIB which then increases risk of appraising your worst healthcare experience as a betrayal. Together, these variables predicted healthcare avoidance.

These findings extend the current literature on understanding predictors of healthcare avoidance. As reported in previous studies, childhood trauma predicts healthcare avoidance [11]. This study extends past research by demonstrating that long-standing and pre-existing beliefs of failure, mistrust of others, and fears about being abandoned (i.e., the impaired autonomy and performance and disconnection and rejection domains of EMS) predict higher levels of healthcare avoidance. One possible interpretation is that patients who hold these beliefs are less likely to trust their healthcare providers which could then lead to higher healthcare avoidance through a less connected and effective patient–provider relationship. In support of this notion, research has documented that early maladaptive schemas have predicted poor trust in relationships [29] and that medical mistrust has predicted healthcare avoidance [12].

Additionally, we found that HIB and betrayal trauma appraisal sequentially mediated the relationship between early maladaptive schemas and healthcare avoidance. It is possible that healthcare providers may perceive patients who hold early maladaptive schemas as more “difficult” to work with collaboratively. For example, patients who have experienced trauma may respond to a clinical encounter in a manner that is protective for themselves but might be more likely to be perceived as “difficult” to work with by providers [30]. In this case, some providers may attribute a patient’s trauma-related behavior to their disposition rather than their situation [31]. Unfortunately, this could lead to strong emotional reactions from providers [32,33] and in some cases, providers may simply end up disliking the patient [34]. These emotional responses from providers could lead to acts of HIB and make it more likely that these patients would appraise their worst healthcare experience as betraying, as was found in our study. In support of this notion, research has highlighted that patients who are labeled as “difficult” to work with may be treated poorly which can increase trauma symptoms [30] and retraumatize the patient [35]. Qualitative studies have also reported that experiencing HIB can lead patients to have strong negative emotional reactions including feeling betrayed, upset, and angry [36], creating a negative care cycle. As found in our study and guided by BITTEN, this set of experiences could then lead to healthcare avoidance. Overall, this possibility highlights the important role the healthcare system has in promoting patients’ future healthcare behavior.

Our findings also suggest multiple groups of patients have additional vulnerability to healthcare avoidance. More specifically, we found that women and People of Color had higher rates of healthcare avoidance. Our findings are consistent with research that suggests minoritized racial and ethnic groups have higher levels of healthcare avoidance [23]. However, our finding that college women reported higher levels of healthcare avoidance than college men is not consistent with the literature as it is typically suggested that men have higher rates of healthcare avoidance as compared to women [24,37]. However, it is important to note that there is some research that suggests that women who hold specific identities such as minoritized racial and ethnic identities and minoritized sexual identities are at greater risk for healthcare avoidance [38]. About 40% of the women in our study identified as a Person of Color. It is also possible that college women were more comfortable than men self-reporting their healthcare avoidance behaviors, as measured in the current study. Future research should continue to identify patient groups who are at high risk for healthcare avoidance. Multi-model, multi-informant strategies should also be developed to assess healthcare avoidance.

## 5. Clinical Implications

This study has important clinical implications for healthcare providers and healthcare systems. For example, given that HIB played a mediating role between early maladaptive schemas and healthcare avoidance, it is important to consider the role the healthcare system has on a patient’s engagement in healthcare avoidance. Our findings, along with previous research, suggest that individual level factors alone cannot fully explain healthcare avoidance. Instead, the treatment received from the healthcare system as a whole can elicit strong emotional reactions from patients, leading them to feel betrayed and upset [36], in turn, reducing their trust in the healthcare system, subsequently lowering their expectations for positive future healthcare experiences [39], and ultimately leading to healthcare avoidance [11].

Given the important role HIB plays in healthcare avoidance, a holistic implication is for healthcare settings to consider and learn from the BITTEN model. The BITTEN model highlights the importance of a trauma-informed care approach [9]. The approach requires providers to realize rates of trauma, recognize trauma symptoms, respond by integrating trauma knowledge into practice, and avoid retraumatizing the patient [9,35]. Healthcare providers using the BITTEN model will need to evaluate a patient’s prior trauma history as well as determine whether they have had previous experience of healthcare institutional betrayal to best address a patient’s current needs and to promote continued engagement. As suggested in our findings, long-standing cognitive and emotional processes, intrinsic to the patients and likely generated by adverse childhood experiences, are also contributing to on-going avoidance behavior. Understanding this might motivate providers to ensure the provision of trauma informed care.

It will also be critical for healthcare professionals to assess for childhood trauma [11] and to understand the maladaptive core beliefs that an individual may hold. As reported in previous studies, childhood trauma predicts healthcare avoidance. It could benefit healthcare providers to routinely assess for childhood trauma, and to consider the maladaptive core beliefs held by patients. Understanding these early maladaptive schemas could help providers build trust and ultimately increase healthcare engagement, especially among those using a trauma-informed care approach.

Assessing for trauma and identifying patients’ early maladaptive schemas might become an important role for the behavioral health provider on the integrated healthcare team [11]. Since behavioral health providers already assist in medication adherence, they may be uniquely positioned to help patients who need to increase their healthcare engagement. Behavioral health providers may be able to meet with patients to help address barriers to healthcare and assist in improving trust between patients and the healthcare setting. This is critical as research suggests that only 27% of physicians across family physicians, psychiatrists, and other medical specialists routinely screen for adverse childhood experiences [40]. Others have suggested that this could be due to a lack of familiarity or frame of reference in asking about prior trauma [11]. However, some studies have suggested that patients feel comfortable and appreciate being asked about trauma [41]. Furthermore, this could help develop a better relationship between the patient and provider [41]. Additional support and training may be needed to move the healthcare system in this direction. Lastly, behavioral health providers may also be able to facilitate continued education opportunities for healthcare providers wanting to learn more about how to assess for trauma and utilize a trauma-informed care approach in their practice.

Another way healthcare systems can address patient’s needs is by repairing the relationship of patients who have experienced HIB. Repairing relationships between patients and the healthcare system, post HIB, could increase a patient’s trust in the healthcare system. Previous reports have suggested that medical mistrust is a significant factor that is associated with healthcare avoidance [12]. Therefore, finding ways to increase trust in healthcare settings could also increase healthcare engagement.

## 6. Limitations

Limitations to this study should be noted. First, while this study consisted of over 1300 participants which afforded considerable power to detect effects, sample sizes remained too small to determine the degree to which the tested models are moderated by a variety of individual characteristics. Yet, we already observed gender and race differences in reports of HIB and healthcare avoidance. Further work with even larger samples will be critical. Additionally, the findings should be replicated in other parts of the country and in other types of settings (private, community college, emerging adults not obtaining higher education). Second, we focused on college students, as many are emerging adults who may be grappling with increased autonomy and independent healthcare-related decision making; however, the extent to which these findings generalize to other populations, including chronic care patients, and individuals from different developmental periods, remains to be determined.

Third, the study design was cross-sectional and relies on college students to self-report their worst healthcare experience via an online survey. Therefore, we cannot claim causality or directionality. We based our models on theory and some temporality can be assumed given the nature of our variables (i.e., EMS develop in childhood; healthcare avoidance is a future-oriented behavior). However, longitudinal designs are best suited for testing predictive models. Moreover, because all variables were assessed via self-report, the obtained associations could be overestimated due to shared variance. Replication of these findings with different designs and with multiple informants will be essential.

Fourth, this study assessed HIB with a measure that is relatively new to the research area. Further measurement development will be important as the internal consistency of our measure was lower than ideal. Another limitation of the utilized HIB measure is that it only measures the presence or absence of acts of HIB; types of affect (i.e., shame and appraisals (self-blame)) during the encounter may also be important to assess [21].

## 7. Future Directions

There are several emerging directions for future research. First, while we tested mediation models in a cross-sectional design, it will be important for future research to test these relationships longitudinally to establish directionality. Furthermore, we focused our research on two specific types of early maladaptive schemas and one form of trauma appraisal. However, healthcare avoidance may result from other forms of early maladaptive schemas as well as from different forms of trauma appraisals. For example, we focused on the EMS disconnection and rejection and impaired autonomy and performance domains as they had the strongest relationships with interpersonal conflict in previous research; however, other EMS domains (e.g., impaired limits and over-vigilance schema), have also been related to interpersonal conflict [16]. Additionally, there are factors that could be driving healthcare avoidance that are not articulated within the BITTEN model, including particular cognitive factors. For example, one’s belief in being able to take care of themselves is associated with lower rates of healthcare avoidance [2]. Future research should continue to explore a wide array of factors that contribute to healthcare avoidance.

It will also be important to explore factors that could increase healthcare engagement. Researchers can examine this in multiple ways. For example, future studies should explore ways to implement a BITTEN model in healthcare settings. Researchers could then empirically test the association between the implementation of the BITTEN model and reduced rates of healthcare avoidance. Additionally, future research should explore the most effective ways to measure and assess HIB. Furthermore, studies could explore how routinely incorporating behavioral health providers into an integrated care setting may influence rates of healthcare avoidance and trust in healthcare settings. Lastly, future studies should explore ways to repair relationships violated by HIB.

Lastly, it will be important to consider settings in which it could be critical to intervene to prevent healthcare avoidance. For example, student health centers may be an ideal intervention spot to repair past HIB. Many students receive care at on-campus student health centers. These centers could routinely assess for past trauma and EMS, using a patient-centered approach to determine needs and expectations, while providing trauma-informed care with an integrated healthcare team.

## 8. Conclusions

Overall, our results suggest that cognitive processes such as EMS predict higher rates of healthcare avoidance, and this relationship is explained through increases in HIB and betrayal trauma during previous problematic healthcare experiences. This highlights the need to identify and address maladaptive cognitive processes that often are generated by adverse childhood experiences as they may activate during problematic healthcare encounters. Our findings also illustrate the role that the healthcare system plays in healthcare avoidance. Therefore, it is important for providers to assess for previous trauma, institute repair post HIB, and increase trust through the enactment of high-quality trauma-informed healthcare, particularly for vulnerable patient groups.

## Figures and Tables

**Table 1:** Sample characteristics. *n* = 1383.

	*n*	%
Race/Ethnicity **		
White	823	59.5
Black/African American	262	18.9
Hispanic/Latino	89	6.4
Asian	99	7.2
Other	55	4.0
American Indian/Alaskan Native	5	0.4
Native Hawaiian/Pacific Islander	5	0.4
Missing	45	3.3
Gender		
Female	845	61.1
Male	488	35.3
Prefer not to say	5	0.4
Prefer to self-describe	6	0.4
Year in College		
Freshmen	661	47.8
Sophomore	430	31.1
Junior	130	9.4
Senior	110	8.0
Chose not to answer	15	1.1

** Note. Adds up to higher *n* than sample as participants could choose more than one response.

**Table 2:** Descriptives.

	*n*	Min–Max	M (*SD*)
Healthcare Avoidance	1177	2.00–12.00	6.00 (2.50)
EMS: Impaired Autonomy Performance	1152	6.00–74.00	31.45 (12.86)
EMS: Disconnection and Rejection	1157	5.00–140.00	57.09 (23.78)
Trauma Appraisal	1135	6.00–35.00	14.39 (7.37)
Healthcare Institutional Betrayal	1383	0.00–11.00	1.41 (1.74)
Age	1251	18.00–65.00	19.86 (3.64)

**Table 3:** Bivariate correlations among focal variables.

	1	2	3	4	5	6
1. Healthcare Avoidance	-					
2. Impaired Autonomy Performance	0.25 ***	-				
3. Disconnection and Rejection	0.28 ***	0.76 ***	-			
4. Healthcare Institutional Betrayal	0.37 ***	0.14 ***	0.19 ***	-		
5. Betrayal Trauma Appraisal	0.37 ***	0.22 ***	0.21 ***	0.43 ***	-	
6. Race	0.05	0.09	0.09 **	−0.09 ***	0.03	-
7. Gender	−0.12 ***	−0.08 **	−0.03	−0.13 ***	−0.07 *	

Note. * *p* < 0.05, ** *p* < 0.01, *** *p* < 0.001; Gender coded 0 = female, 1 = male; Race coded 0 = White and 1 = People of Color.

**Table 4:** Regression coefficients, standard errors, and model summary information for EMS: impaired performance and autonomy and disconnection and rejection predicting healthcare avoidance serial multiple mediator.

Antecedent	Step One			Step Two			Final Model		
	M_1_ (HIB)			M_2_ (BTA)			Y (HAV)		
	*b*	*SE*	*p*	*b*	*SE*	*p*	*b*	*SE*	*p*
X (EMS-IAP)	0.02	0.00	0.000	0.09	0.02	0.000	0.03	0.01	0.000
M_1_ (HIB)	—	—	—	1.71	0.11	0.000	0.34	0.04	0.000
M_2_ (BTA)	—	—	—	—	—	—	0.07	0.01	0.000
Gender	−0.61	0.11	0.000	0.04	0.42	0.923	−0.24	0.14	0.082
Race	−0.37	0.11	0.001	0.76	0.42	0.067	0.28	0.14	0.039
Constant	1.46	0.15	0.000	8.27	0.60	0.000	3.31	0.22	0.000
*R* ^2^	*R*^2^ = 0.05			*R*^2^ = 0.22			*R*^2^ = 0.23		
	F(3,1066) = 21.20, *p* = 0.000			F(4,1065) = 73.22, *p* = 0.000			F(5,1064) = 62.30, *p* = 0.0000		
Antecedent	Step One			Step Two			Final Model		
	M_1_ (HIB)			M_2_ (BTA)			Y (HAV)		
	*b*	*SE*	*p*	*b*	*SE*	*p*	*b*	*SE*	*p*
X (EMS-Disc-Rej)	0.01	0.00	0.000	0.04	0.01	0.000	0.02	0.00	0.000
M_1_ (HIB)	—	—	—	1.70	0.12	0.000	0.32	0.04	0.000
M_2_ (BTA)	—	—	—	—	—	—	0.08	0.01	0.000
Gender	−0.65	0.11	0.000	−0.13	0.42	0.759	−0.30	0.14	0.032
Race	−0.43	0.11	0.000	0.72	0.42	0.085	0.23	0.14	0.103
Constant	1.27	0.15	0.000	8.97	0.58	0.000	3.28	0.21	0.000
*R* ^2^	*R*^2^ = 0.07			*R*^2^ = 0.21			*R*^2^ = 0.23		
	F(3,1072) = 29.54, *p* = 0.000			F(4,1071) = 70.30, *p* = 0.000			F(5,1070) = 64.55, *p* = 0.000		

Note. IAP = Impaired Autonomy and Performance, Disc-Rej = Disconnection and Rejection HIB = Healthcare Institutional Betrayal, BTA = Betrayal Trauma Appraisal, HAV = Healthcare Avoidance.

## Data Availability

Data are available upon request.
